# Cholera Toxin B: One Subunit with Many Pharmaceutical Applications

**DOI:** 10.3390/toxins7030974

**Published:** 2015-03-20

**Authors:** Keegan J. Baldauf, Joshua M. Royal, Krystal Teasley Hamorsky, Nobuyuki Matoba

**Affiliations:** 1 Department of Pharmacology and Toxicology, University of Louisville School of Medicine, Louisville, KY 40202, USA; E-Mail: keegan.baldauf@louisville.edu; 2 Owensboro Cancer Research Program of James Graham Brown Cancer Center at University of Louisville School of Medicine, Owensboro, KY 42303, USA; E-Mails: joshua.royal@ocrp.org (J.M.R.); krystal.hamorsky@ocrp.org (K.T.H.); 3 Department of Medicine, University of Louisville School of Medicine, Louisville, KY 40202, USA

**Keywords:** *Vibrio cholerae*, cholera toxin B subunit, vaccine adjuvant, anti-inflammatory

## Abstract

Cholera, a waterborne acute diarrheal disease caused by *Vibrio cholerae*, remains prevalent in underdeveloped countries and is a serious health threat to those living in unsanitary conditions. The major virulence factor is cholera toxin (CT), which consists of two subunits: the A subunit (CTA) and the B subunit (CTB). CTB is a 55 kD homopentameric, non-toxic protein binding to the GM1 ganglioside on mammalian cells with high affinity. Currently, recombinantly produced CTB is used as a component of an internationally licensed oral cholera vaccine, as the protein induces potent humoral immunity that can neutralize CT in the gut. Additionally, recent studies have revealed that CTB administration leads to the induction of anti-inflammatory mechanisms *in vivo*. This review will cover the potential of CTB as an immunomodulatory and anti-inflammatory agent. We will also summarize various recombinant expression systems available for recombinant CTB bioproduction.

## 1. Introduction

### 1.1. Cholera

Cholera is a highly contagious acute dehydrating diarrheal disease caused by *Vibrio cholerae*. There are over 200 serogroups of *V. cholerae* known to date; however, only two (O1 and 139 serotypes) are responsible for the vast majority of outbreaks [1,2]. The pathology of cholera results from *V. cholerae* colonization in the small intestine and subsequent production of the cholera toxin (CT).

*V. cholerae* are found in coastal waters and deltas due to their preference for salinity in water; however under proper conditions (warm and sufficient nutrients), *V. cholerae* can grow in low salinity environments [3]. Natural disasters (e.g., floods, monsoons, and earthquakes) and poor sanitation are major players in the spread of cholera epidemics. Symptomatic individuals can shed the organism from 2 days to 2 weeks after infection and recently shed organisms (5–24 h after shedding) have hyperinfectivity; in this state the infectious dose is 10 to 100 times lower than non-shed organisms (~10^6^ bacteria) [4,5]. This can lead to the rapid spread of cholera in densely populated areas without proper management of patients and their waste.

The most common symptom of cholera is a life-threatening amount of watery diarrhea, causing an extreme loss of water, up to 1 L per hour, which can lead to death within hours of the first onset of symptoms if left untreated [3]. The diarrhea is usually painless and not accompanied by the urge to evacuate the bowels. Early in the illness, vomiting can be a common symptom as well.

Cholera is considered endemic in over 50 countries, but it can manifest as an epidemic, as has recently been the case in Haiti (2010–present), a country previously not exposed to cholera [6,7,8]. Reported world incidences of cholera increased from 2007 until a peak of approximately 600,000 cases in 2011 [9]. In 2012, the number of reported cases decreased to approximately 245,000 with 49% of the cases resulting from the ongoing outbreak in Haiti and the Dominican Republic. However, the World Health Organization (WHO) estimates the actual global burden of the disease to be between 3 and 5 million cases per year and 100,000 to 130,000 deaths per year [10]. Additionally, a more virulent strain of *V*. *cholerae* O1 is making inroads in Africa and Asia [11]. The WHO suggests there should also be concern for the spread of antibiotic‑resistant strains of *V. cholerae*. This has already been shown with *V. cholerae* O139 and some isolates from *V. cholerae* O1 El Tor, which have acquired resistance traits for co-trimoxazole and streptomycin [3]. It is clear that cholera, despite its long history, is still an emerging disease that is necessary to combat.

### 1.2. CT

**Figure 1:**
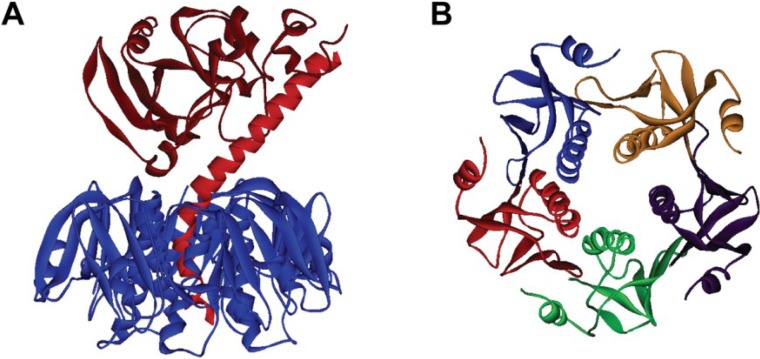
Cholera toxin (CT) crystal structure. (**A**) CT (side view; Protein Data Bank [PDB] ID: 1XTC). The CTA subunit is shown in red (CTA1 in dark red and CTA2 in light red) and the CTB subunit is shown in blue; (**B**) CTB (top view; PDB ID: 1XTC with CTA subunit removed). Each monomer of the B subunit is show in a different color. Images were created in Accelrys Discovery Studio Visualizer 2.5.

CT produced by *V. cholerae*, is the main virulence factor in the development of cholera. The molecular characteristics of CT and its toxic effects in humans have been well characterized [12,13,14]. CT is an 84 kD protein made up of two major subunits, CTA and CTB [15,16] (Figure 1). The CTA subunit is responsible for the disease phenotype while CTB provides a vehicle to deliver CTA to target cells. CTA is a 28 kD subunit consisting of two primary domains, CTA1 and CTA2, with the toxin activity residing in the former and the latter acting as an anchor into the CTB subunit [17]. The CTB subunit consists of a homopentameric structure that is approximately 55 kD (11.6 kD monomers) and binds to the GM1-ganglioside; found in lipid rafts, on the surface of intestinal epithelial cells [13]. The exact mechanism of delivering CTA1 into the intracellular space is still not fully resolved; however, the current understanding is that CT is endocytosed and travels through a retrograde transport pathway from the Golgi apparatus to the endoplasmic reticulum (ER) [12,13,14,17,18]. Recently, it has been shown that CT can also move from the apical to basolateral surface of epithelial cells via transcytosis, enabling transport of whole CT through the intestinal barrier [19]. CTA is dissociated from CTB after the toxin reaches the ER and translocated to the cytosol via the ER-associated degradation pathway [15]. Intoxication occurs when CTA1 enters the cell cytosol and catalyzes the ADP ribosylation of adenylate cyclase, which leads to increased intracellular cAMP. This increase in intracellular cAMP results in impaired sodium uptake and increased chloride outflow, causing water secretion and diarrhea [12,17].

### 1.3. Current Vaccines

The emergence of a more virulent strain of *V. cholerae*, coupled with the increasing number of endemic and newly exposed countries suggests a growing need for a consistent vaccination strategy. Currently, there are two WHO pre-qualified vaccines for cholera: Dukoral^®^ (SBL Vaccin AB, Stockholm, Sweden) and Shanchol^®^ (Shantha Biotechnics Limited, Basheerbagh, India). Dukoral^®^ contains killed *V. cholerae* (Inaba and Ogawa serotypes of *V. cholerae* O1) and recombinant (r) CTB, while Shanchol^®^ contains the killed *V. cholerae* (serogroups O1 and O139) [20]. Due to the cross‑reactivity of anti-CTB antibodies to heat labile enterotoxin (LTB), Dukoral^®^ is also effective against enterotoxigenic *Escherichia coli* (ETEC), an advantage not offered by Shanchol^®^. On the other hand, Shanchol^®^ is a less expensive cholera vaccine than Dukoral^®^ because the latter includes costs related to rCTB, *i.e.*, recombinant production, a buffer to neutralize stomach acid to prevent rCTB degradation and additional storage space and logistics. In a vaccination cost analysis study performed in 2012, it was found to cost approximately US$10 to purchase two doses of Dukoral^®^ and approximately US$3 to deliver those doses [21]. However, these costs could be reduced by developing cost-effective rCTB production methods (see below) and formulating the vaccine in a solid oral dosage form able to pass through the stomach and dissolve in the small intestine [22].

Interestingly, a field trial performed in 1985 suggests that a whole cell-killed vaccine with CTB (WCB) may be more efficacious than a whole cell-killed vaccine without CTB (WC) [23]. Children 2 to 10 years old were almost completely and significantly protected (92%) from cholera after 3 vaccinations with WCB compared to a non-significant 53% protection for WC for the first six months after vaccination. Hence, children were far better protected with the CTB-containing vaccine. In older populations (>10 years old) both vaccines showed similar protective efficacy over 6 months; the WCB vaccine protected 77% of the adults compared to 62% with the WC vaccine. Additionally, perhaps most importantly, the WCB vaccine significantly protected against severe cholera episodes (89% protective) *versus* no significant protection by the WC vaccine (44% protective). Lastly, within approximately the first 6 months following vaccination, the WCB vaccine significantly protected the recipients while WC vaccine recipients lost protective efficacy approximately 3 months after vaccination. This short-term enhanced protection could provide a significant implication for a reactive vaccination strategy to contain outbreaks.

The same population was also tracked for three years following vaccination and differences between WCB and WC vaccination were further elucidated [24]. Again, it was found that 2–5 year old children, who received all three vaccine doses, were significantly protected when receiving the WCB vaccine for up to 2 years following vaccination when compared to the placebo group. At no point was WC vaccine significantly protective of the 2–5 year old cohort in this study. For up to 3 years following vaccination both WCB and WC protected study participants over the age of 5. Additionally, the number of doses needed to see strong protection against cholera was another point of differentiation. WCB vaccination required two doses to provide significant protection while the same level of protection was not achieved with the WC vaccine until a third dose was administered. It should be noted that WCB contains non-recombinant CTB (purified from CT) and thus should not be confused with the currently available Dukoral^®^, which contains rCTB.

In this regard, a more recent work has been performed to evaluate the protective efficacy of Dukoral^®^ in adults and children [25]. The study by Alam *et al.*, divided children into 2 groups: young (median age 5) and older (median age 10) and had an adult group with a median age of 32. Significant antibody responses in all groups were seen 3 days following the first dose in all study groups and continued to day 42 in all groups. However at day 90, the next time point in the study, both groups of children lost the antibody response while the adult antibody response persisted until at least 270 days following the second vaccination. Additionally, a 2005 study in Mozambique showed that an rCTB whole cell-killed vaccine was able to protect at similar levels of the WCB vaccine used in Bangladesh [26]. The results from this study also confirmed that the vaccine containing rCTB may have improved protection in severe cases of cholera. Confounding these results, a field trial performed in Peru in 1994 is often reported as having negative results (increased cholera infection) in rCTB vaccine recipients [27]. However, the study did report positive protection after a booster third dose was given just prior to the start of the next cholera outbreak season in Peru. Additionally, this study evaluated only two time points, 1 year and 2 year protection, which could have overlooked the early protection (<6 months after vaccination) observed previously with WCB [28]. Lastly, the fact that a single booster provided protection during the second year of the study suggests that an rCTB containing vaccine does in fact protect against cholera outbreaks.

Shanchol^®^ has been studied in both Bangladesh and Haiti; participants in both studies showed strong immune responses to the two dose vaccine regimen [20,29]. In 2012, Shanchol^®^ was used in an outbreak in Guinea and found to be effective in protecting adults from cholera infection [30]. These findings were thought to be in line with results seen with Dukoral^®^, but there was no rCTB vaccine group in this study to compare to. An advantage to Shanchol^®^ is that it has been tested in children as young as 1 year old and protection has been noted in this young population [29]. The lack of a large scale study comparing Shanchol^®^ and Dukoral^®^ makes any comparison difficult.

A recent paper may help elucidate the potential benefit of including rCTB in any cholera vaccine. Although mice do not develop cholera, a model of pulmonary *V. cholerae* infection has recently been established [31]. In this model, severe pneumonia was induced in mice and was found to be fatal within several days of inoculation with *V. cholerae*. Interestingly, mice vaccinated intranasally, twice with Dukoral^®^ prior to *V. cholerae* challenge, were significantly protected compared to controls. Unvaccinated animals died within 24 h of the challenge while none of the mice vaccinated died for up to 7 days following challenge. Notably, Dukoral^®^ without rCTB showed no protection in this model, while protection was restored upon inclusion of rCTB. These results provide unequivocal evidence that rCTB is essential in protecting mice from the lethal pneumonia induced by *V. cholerae* infection. Coupled with the earlier findings with WCB vaccines in the field trial, it is suggested that, in the case of cholera outbreaks, vaccines containing rCTB may provide immediate benefit to vaccine recipients that would not be seen in rCTB-free vaccines.

## 2. CTB as a Vaccine Adjuvant

In addition to its toxic properties, CT is also known to have strong mucosal immunogenic properties that have been investigated for beneficial use as well as inducing an allergic response in animal models [32,33,34,35,36,37]. CT has also been shown previously to have adjuvant potential when incorporated into mucosal vaccines [38,39,40]. However, the toxicity of CT made its use in humans undesirable and work now focuses on removing the toxicity from the molecule while maintaining the adjuvant effect. The CTB subunit was previously shown to induce an immune response without the toxicity associated with the CTA subunit [41]. CTB has proven to be a strong adjuvant to uncoupled antigens when administered via the nasal route but less so when administered orally [15,42,43]. However, the nasal route of administration is not preferred due to the potential risk for developing Bell’s palsy [44,45,46]. Fortunately, it was found that by coupling the antigen to CTB, a much stronger response is achieved via the oral administration route [47]. We should also point out that the adjuvant potential of CTB has also been shown in large animal models, indicating that the adjuvant potential is scalable to higher species [48,49,50]. The utility of CTB becomes apparent when looking at the various disease states in which it has been used as an adjuvant: bacterial and viral infections, allergy, and diabetes have been targeted [51,52,53]. Also, an interesting approach to resolving cocaine addiction has been attempted by binding rCTB to succinylnorcocaine, which has been tested in a Phase IIb randomized double-blind placebo-controlled trial [54,55]. The hypothesis behind the vaccine was that the anti-cocaine antibodies may block the uptake of cocaine in the brain from the blood. While the results were inconclusive, with only ~40% of participants achieving inhibitory antibody concentrations in the blood, this study shows potential utility of CTB-based vaccines in addiction therapy.

For a general overview of the work on CTB as a vaccine adjuvant, readers are referred to thorough reviews published previously [41,56,57]. For this review we will focus on some findings not addressed in these previous reviews.

### 2.1. CTB-Based Immunogens against Bacterial Pathogens

Development of vaccines against several bacterial pathogens has been attempted recently by conjugating antigens to CTB to induce immune responses against the bacteria. *Helicobacter pylori* is a bacterium that infects greater than 50% of the world population and can cause a variety of gastrointestinal diseases [58]. Specifically, *H. pylori* urease, a two subunit enzyme, has been targeted by linking both subunits (UreA and UreB) of the enzyme to CTB. Guo *et al.* described a fusion protein of rCTB with the B cell epitope of UreA (denoted rCTB-UA) that was expressed in *E. coli* [58]. In a mouse immunization experiment they found that rCTB-UA could induce antibodies to UreA and UreB proteins, which inhibited the activity of *H. pylori* urease. In a follow up paper, the group showed prophylactic and therapeutic dosing with rCTB-UA could protect mice from *H. pylori* infection [47]. This work has resulted in a second generation epitope vaccine (rCTB-UE) which not only consists of the original B cell epitope but a T helper cell epitope from both UreA and UreB [51,59]. In a Mongolian gerbil model of *H. pylori* infection, rCTB-UE protected against infection and decreased inflammation in the gastric tissue (inflammatory cytokines and histology) [59]. Additionally, the paper showed that the immune-protective mechanism of rCTB-UE was related to the upregulation of microRNA-155, which led to the activation of T helper (Th)1 and B cell immune responses against *H. pylori* infection. Meanwhile, Kono *et al.* showed protection from a fatal systemic infection of *Streptococcus pneumonia* in 10 day old mouse pups immunized via breast milk from mothers [60]. The mothers were intranasally immunized with Pneumococcal surface protein A (PSPA) and CTB and the anti-PSPA antibodies were present in serum and breast milk of the mothers. Through breast feeding, the offspring were protected from *S. pneumonia* infection. This study provided an important finding that mucosal immunization of a female population with vaccines containing CTB may be able to protect their offspring during early stages of life, when they are most vulnerable to respiratory diseases.

### 2.2. CTB-Based Immunogens against HIV

Viral pathogens have also been targeted by CTB-based vaccine development research. Given that CTB has the ability to induce potent mucosal humoral immune responses, perhaps the best opportunity to exploit CTB may be found in vaccines against mucosally transmitting viruses, such as human immunodeficiency virus (HIV-1). Indeed, a number of studies have used CTB as a mucosal adjuvant component of experimental HIV-1 vaccines [61,62,63,64,65,66,67].

Over the past decade, we reported a series of studies demonstrating that rCTB-MPR_649–684_, a rCTB fusion protein displaying a peptide spanning the HIV-1 gp41 membrane proximal region, is capable of inducing gp41-binding antibodies in mice and rabbits [61,68,69,70,71]. These antibodies efficiently blocked transcytosis of primary HIV-1 isolates in a human tight epithelial model, suggesting that rCTB-MPR_649–684_ protein may provide an effective prophylactic vaccine preventing HIV-1 mucosal transmission [61,69,70]. In a separate study, CTB was co-administered with a plasmid generated from an envelope protein (gp145_5m_) of HIV-1 intramuscularly to mice [64]. The immune response by intramuscular dosing with gp145_5m_ and CTB was significantly enhanced when compared to gp145_5m_ alone. This study confirms that CTB, while an effective adjuvant via the nasal or oral administration routes, can also be considered for intramuscular dosing vaccine regimens to enhance the immune response. Meanwhile, Maeto *et al.* evaluated if supplementing a DNA plasmid expressing an HIV-1 Env and Interleukin-12 (IL-12) with CTB could enhance the immune response after intranasal immunization in mice [63]. IL-12 had previously been reported to enhance an antigen-specific immune response by the intranasal vaccination route [72]. In this study, not only did the combination enhance the immune response to the HIV-1 Env antigen but also significantly decreased the concentration needed to trigger Interferon (IFN)-γ, a Th1 cytokine, production by 3 times. HIV-specific CD8 responses in spleen and genital tract and genito-rectal draining lymph nodes were effectively improved, showing cytotoxic T cell responses with higher avidity, polyfunctionality and cytolytic activity. Hence, the results indicate that a greater adjuvant effect can be achieved when CTB is co-administered with another adjuvant.

### 2.3. Novel CTB-Based Vaccine Delivery and Antigen Conjugation Methods

In the majority of previous studies, CTB has been administered directly to mucosal surfaces via the intranasal or oral routes. In contrast, Hu *et al.* recently reported a novel approach of delivering CTB to the mucosa. In this study, they orally administered genetically engineered *Bacillus subtilis* to mice and guinea pigs, which expressed multiple epitopes of the foot-and-mouth disease virus and rCTB [73]. This method induced a significantly stronger immune response compared to the commercially available vaccine in the gut and lung, although upon viral challenge, the commercial vaccine provided slightly better protection in immunized animals.

In addition to mucosal routes of administration, CTB has been used as a component of a skin patch to vaccinate against hepatitis B virus in mice. The study was aimed at showing that transcutaneous immunization, involving microneedles which penetrate the stratum corneum without contacting nerves followed by applying a medicated patch to the area, could effectively produce antibodies against the hepatitis B surface antigen (HBsAg). CTB showed the ability to not only enhance the immune response against HBsAg but also extend the duration of protection through the transcutaneous immunization route [74]. Combined with results of other studies using a similar strategy [75,76,77,78], there is now a compelling reason to explore the development of transcutaneous vaccines including CTB as an adjuvant.

While antigen-CTB coupling has been most commonly achieved by chemical crosslinking to specific functional groups of amino acid residues or genetic fusion to the *N*- or *C*-terminus of CTB, an alternative approach has been seen in the literature that uses the CTA2 domain to link antigens to CTB [52,79,80]. For example, this approach was used for a vaccine against West Nile virus, in which the domain III (DIII) region of the virus was used as the antigen genetically fused to the CTA2 domain (see Figure 1). The DIII-CTA2 protein was co-expressed with rCTB to form a chimeric CT-like molecule, DIII-CTA2/B [52]. Intranasal delivery of DIII-CTA2/B in mice produced DIII-specific antibodies that could trigger complement-mediated killing. Although not as heavily studied as conventional CTB *C*/*N*‑terminal fusion methods, the CTA2/B strategy may provide a useful means to develop a vaccine comprising a relatively large antigen.

Lastly, CTB has been incorporated into other alternative drug delivery systems such as liposomes, microspheres and nanoparticles. Harokopakis and colleagues found that coating liposomes with rCTB enhanced the immune response against the saliva-binding region of *S. mutans* AgI/II adhesin [81]. O’Hagan *et al.*, encapsulated rCTB in poly(lactide-co-glycolide) microparticles, which showed comparable humoral immunogenicity with CTB admixed with CT upon oral administration in mice [82]. In a more recent example, a DNA vaccine for cholera (pVAX-ctxB) encapsulated in microspheres, allowing the vaccine to pass through the acidic environment of the stomach, has shown the ability to generate an immune response in mice [83].

## 3. CTB in Inflammation

Besides the mucosal vaccine adjuvant activity summarized above, recent studies have revealed that CTB can also induce anti-inflammatory and regulatory T cell responses. Indeed, the protein was shown to suppress immunopathological reactions in allergy and autoimmune diseases (reviewed in: [57]). In a mouse model, the airway administration of CTB ameliorated experimental asthma [84]. Furthermore, the anti-inflammatory and immunoregulatory effects of CTB are effectively conferred on bystander protein antigens that are chemically or genetically linked to CTB; oral administration of rCTB chemically cross-linked to a peptide from the human 60 kD heat shock protein was shown to mitigate uveitis of Behcet’s disease in a Phase I/II clinical trial [85]. Meanwhile, rCTB was also shown to mitigate the intestinal inflammation of Crohn’s disease in mice and humans [57]. Below, we will highlight some of these and a few other recent findings regarding CTB as an anti-inflammatory agent.

### 3.1. CTB’s Anti-Inflammatory Activity in Various Inflammatory Diseases

Type 1 Diabetes Mellitus induces cellular oxidative stress which leads to chronic inflammation and secondary effects such as: atherosclerosis, blindness, and stroke [86]. CTB has been used to target multiple anti-inflammatory agents that alone were either short lived or could not effectively induce an immune response. An example of this comes from Odumosu *et al.*, who fused glutamic acid decarboxylase (GAD) to rCTB (GAD-rCTB) and showed suppression of dendritic cell activation in human umbilical cord blood isolated dendritic cells [87]. Dendritic cells are often implicated in islet β-cell loss in Type 1 Diabetes so this presents an attractive therapeutic option. Additionally, the group showed that pro-inflammatory cytokines, IL-12 and IL-6, were down-regulated while IL-10 was significantly increased *in vitro* using dendritic cells. Another study was performed incorporating GAD with rCTB and a recombinant vaccinia virus (rVV) by Denes *et al.*, which co-administered the rVV-rCTB-GAD generated in their lab with Complete Freund’s adjuvant (CFA) to see if multiple adjuvants could further enhance the immune response to the vaccine [88]. Vaccination with both rVV-rCTB-GAD alone and CFA alone showed some measureable protection in the NOD mouse model of diabetes compared to control animals given PBS at approximately 39 weeks of age. However, when rVV‑rCTB-GAD and CFA were combined, hyperglycemia was delayed further to 43 weeks of age. Overall, the study showed by combining the vaccines, NOD mice could be protected from hyperglycemia and pancreatic islet inflammation better than either vaccine alone.

CTB had previously been shown to protect against uveitis resulting from Behcet’s disease in a clinical trial performed in 2004 [85]. This work linked a T cell proliferative peptide (p336–351) to rCTB, which conferred protection on 5 of 8 patients following withdrawal of all immunosuppressive drugs. Other CTB conjugates have also been evaluated in a mouse model of uveitis and shown promise more recently [89]. Shil and colleagues delivered two components of the Renin-angiotensin system (RAS) to the retina, ACE2 and Ang-(1–7) by fusing them to rCTB and administering them orally to mice. Protection was noted by decreased inflammatory cytokines (e.g., IL-6, IL-1β, and TNF-α) and inflammation scoring. Additionally, these components were significantly elevated in the retina of the mice. This study showed that CTB can also be used as a delivery system to inflamed tissue and not just to enhance an immune response.

Atherosclerosis, an inflammatory condition, has recently become a target for rCTB fusion proteins [90,91,92]. In 2010, a mouse model of atherosclerosis showed protection by nasal administration of an rCTB fusion protein (p210-CTB) [91]. The p210 portion is derived from the apolipoprotein B-100 (ApoB100) peptide sequence as an alternative to a low density lipoprotein. Indeed this vaccination strategy reduced atherosclerotic lesion formation and provided some clues to mechanism. IL-10 was significantly upregulated by p210-CTB, while transforming growth factor-β (TGF-β) was not, which led the authors to hypothesize that T regulatory 1 (T_R_1) cells may be responsible for the protection. However, FoxP3 was upregulated thus the authors could not rule out some level of protection from the FoxP3^+^ T regulatory cell population as well. Interestingly, T_R_1 cells are believed to play a more important role when immunity is conferred through nasal administration [93]. A second rCTB-linked protein targeting both ApoB100 and cholesteryl ester transfer protein (implicated in atherosclerosis pathogenesis) was explored more recently, in a proof of concept study, in which antibodies were detected in mouse serum to the target proteins [92]. In this study, the route of administration was by foot pad injection, so it will be interesting to see if altering the route of administration will have impacts on the efficacy and/or mechanism of protection from atherosclerosis.

Liver inflammation and fibrosis were also significantly blunted by an intranasal administration of a rCTB-Sm-p40 egg antigen immunodominant peptide fusion in mice following infection with *Schistosoma mansoni*, which results in schistosomiasis [94]. This protection was associated with a significant increase in TGF-β in the mesenteric lymph node (MLN) CD4 T cells and granuloma cells. The studies on atherosclerosis and this study suggest that CTB may have a compartmentalized effect on TGF-β production in tissues, since both conjugates were administered intranasally, yet only the MLN CD4 T cells and liver granuloma cells showed elevated TGF-β.

Organ transplantation can lead to rejection through inflammation. In a rat model of kidney transplantation, an anti-inflammatory D-amino acid decapeptide, RDP58, chemically conjugated to CTB was shown to enhance the survival time compared to the therapeutic compound alone [95]. Allergic inflammation in mouse airways has also been shown to be reduced by CTB administration, not only in a preventative sense but also in mice that have already been sensitized to airway inflammation [84].

Lastly, CTB has shown in animal models as well as clinical trials to be effective in decreasing inflammation in Inflammatory Bowel Disease (IBD). IBD is subcategorized into Crohn’s disease and ulcerative colitis. In 2001, Boirivant *et al.* showed that oral administration of rCTB protected against Trinitrobenzene Sulfonic Acid (TNBS) induced intestinal inflammation, which is a mouse model resembling Crohn’s disease [96]. This finding was further explored to reveal that IL-12 and IFN-γ were significantly downregulated by rCTB administration in TNBS induced colitis [97]. In addition, rCTB inhibited both STAT-4 and STAT-1 activation and downregulated T-bet expression. These results showed a possible mechanism for protecting against inflammation by inhibiting Th1 cell signaling. The protection seen in the TNBS colitis model was confirmed in a human clinical trial, in which rCTB significantly decreased inflammation in mild to moderately active Crohn’s disease [98]. However, IFN‑γ did not correlate with the reductions in Crohn’s disease activity index in the patients. This might suggest that CTB reduced inflammation in humans through more than inhibition of Th1 cell signaling. On the other hand CTB’s effect in ulcerative colitis, which is another form of IBD involving inflammatory signaling and pathogenesis that is different from that of Crohn’s disease, is currently not known. As noted earlier in the atherosclerosis and liver fibrosis studies, CTB’s anti-inflammatory potential seems to be mediated by different pathways despite having the same route of administration. In this regard, it is of particular interest to investigate whether oral administration of CTB may have therapeutic potential in both Crohn’s disease and ulcerative colitis.

### 3.2. Recombinant or Non-Recombinant CTB: Conflicting Results of CTB’s Anti-Inflammatory Activity in in Vitro Experiments

While a number of studies have reported the anti-inflammatory activity of CTB *in vitro* and *in vivo*, the quality of the CTB used in those studies has not been consistent, which may have had a significant impact on the results of some of those studies. Hence, before concluding this section, we would like to point out the potential influence that the quality of the CTB may have on the outcome of anti-inflammatory studies, particularly those using cell culture experiments.

**Figure 2:**
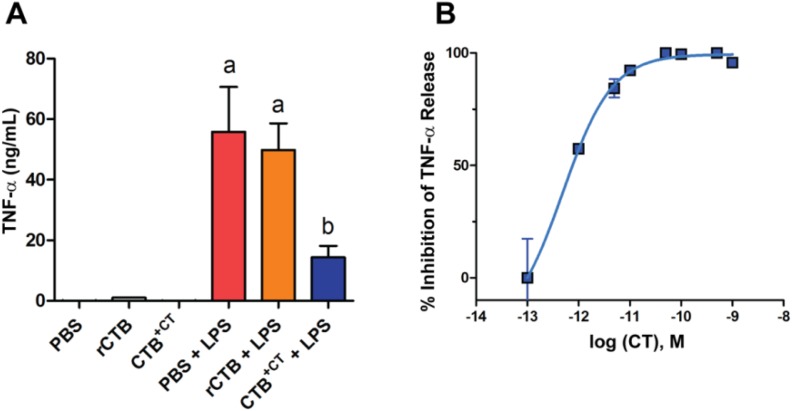
CT, not rCTB, inhibits the release of TNF-α by Raw 264.7 cells stimulated with LPS. (**A**) Commercial non-recombinant CTB containing a trace amount of CT (CTB^+CT^) significantly reduces the production of TNF-α due to LPS stimulation. Raw 264.7 cells were pretreated with 10 μg/mL rCTB (produced in *E. coli* [100]), CTB^+CT^ (Sigma‑Aldrich, St. Louis, MO, USA; catalog no. C9903), or PBS, and a final concentration of 1 μg/mL LPS was added and incubated for 24 h. TNF-α levels in cell supernatants were determined using a commercial ELISA kit (eBioscience, San Diego, CA, USA). Data represent the mean ± SEM (*n* = 4). a: *p* < 0.001, compared to PBS; b: *p* < 0.05, compared to PBS + LPS and rCTB + LPS (one-way ANOVA with Bonferroni multiple comparison tests); (**B**) Picomolar levels of CT inhibit the production of TNF-α. Raw 264.7 cells were pretreated for 2 h with varying concentration of CT, and a final concentration of 0.1 μg/mL LPS was added and incubated for 6 h. The 50% inhibitory concentration (IC_50_) of CT was determined by non-linear regression analysis (GraphPad Prism 5.0, GraphPad Software, Inc., La Jolla, CA, USA) to be 0.49 pM. Data represent the mean ± SEM (*n* = 2). The TNF-α level of PBS + LPS was 4516.8 ± 791.1 pg/mL (mean ± SEM; *n* = 2).

Many of the early studies have used non-recombinant CTB obtained from a commercial source, which is prepared from the CT holotoxin by chemical dissociation of CTA and CTB subunits. As a result, there is a trace amount of CT and CTA subunit remaining in the CTB product [99]. In a conventional *in vitro* assay using the murine macrophage cell line RAW264.7, we found that a commercial CTB product (Sigma-Aldrich, St. Louis, MO, USA; C9903), which contains ≤0.5% of CT according to the datasheet provided, significantly inhibited the production of TNFα induced by lipopolysaccharides (LPS), while rCTB produced in *E. coli* (purified to >95% homogeneous pentamer, with <0.003 endotoxin unit/µg) failed to show such an effect (Figure 2A) [100]. Notably, in this assay picomolar concentrations (<10 ng/mL) of CT exerted strong anti-inflammatory activity (Figure 2B). These results indicate that the trace amount of CT contamination in non-recombinant CTB products could have a major impact on results generated in similar assay systems. Hence, care should be taken when choosing the source of CTB for anti-inflammatory studies. It should be noted that some of the groundbreaking studies showing CTB’s anti-inflammatory activity outlined above, including human clinical studies, have used rCTB. Consequently, there is compelling evidence for the immunotherapeutic potential of rCTB in various inflammatory disorders.

## 4. rCTB Production Methods

Given that CTB exerts strong mucosal immunomodulatory effects and rCTB is currently used in the WHO-prequalified oral cholera vaccine Dukoral^®^ (see above), the protein has provided an attractive target for various recombinant production platforms. These include prokaryotic cells such as genetically modified *V. cholerae*, *E. coli*, *Bacillus* and *Lactobacillus*, as well as eukaryotes ranging from yeast cells to multicellular organisms such as silkworms and plants (Table 1) [100,101,102,103,104,105,106,107,108,109,110,111,112,113,114,115,116,117,118,119,120,121,122,123,124,125,126]. In cell culture systems rCTB is produced in fermenters and bioreactors [102,103,104,105,106,107,108]. Alternatively, in plant expression systems, rCTB is expressed in whole plants grown in controlled growth rooms or greenhouses [100,101,112,113,114,115,116,117,118,119,120,121,122,123,124,125,126].

Plant-based production of rCTB has been approached from two different angles. One approach is to vaccinate individuals with raw or minimally processed edible tissues of transgenic plants expressing rCTB (edible vaccines). For example, carrots, rice, tomatoes, potatoes and maize have been engineered to produce rCTB using transgenic technologies [101,112,113,114,115,116,117,118,119,121,122,123,124,125]. Among these, rice has provided the most advanced platform thus far towards an edible cholera vaccine. Yuki and colleagues have developed a transgenic rice expressing rCTB in the seed endosperm and showed that oral administration of the rice seeds induced CT holotoxin-neutralizing antibodies in mice and non-human primates [126]. No major side effects, including an IgE response to rice endogenous proteins, were observed. Interestingly, however, rCTB was shown to be *N*-glycosylated upon expression in plant cells. To avoid this unique post-translational modification, the same group created a mutant of CTB by replacing the corresponding Asn residue to Gln, and showed that the mutant expressed in transgenic rice endosperm was similarly effective to the original rice-based vaccine in mice and macaques [115]. These studies suggest that the rice-based experimental vaccine may provide a cost-effective oral cholera vaccine. It remains to be seen whether the approach of using edible plant tissue to deliver vaccines could be feasible from regulatory and public acceptance standpoints.

**Table 1:** rCTB Production Systems.

System	Expression Host	Functional Evaluation	Mode of Expression	CTB Yield	Purification	Reference
**Bacterial fermentation**	*V. cholerae*	Affinity for GM1-ganglioside confirmed (GM1-ELISA) and immunogenic in mice	Expression plasmid: (pML-LCTB*tac*2) transformation	1g/L culture	Affinity chromatography (lyso-GM1 ganglioside Spherosil column)	[102]
	*E. coli*	Affinity for GM1-ganglioside confirmed (GM1-ELISA)	Expression plasmid: pQE30 transformation	9 mg/L culture	IMAC* Purification and membrane-filtration	[103]
		Detected by anti-CT antibody (Western Blot)	Expression plasmid: pAE_*ctx*B transformation	1.2g/L culture	Centrifugation	[104]
		Affinity for GM1-ganglioside confirmed (GM1-ELISA)	Expression plasmid: pTG8148 transformation	1 g/L culture	Cation exchange Chromatography (S-Sepharose FF column)	[105]
		Detected by anti-CT antibody (Western Blot)	Expression plasmid: pGEM-T-*ctx*B transformation	80 mg/L culture	Centrifugation	[106]
	*Lactobacilli*	Affinity for GM1-ganglioside confirmed (GM1-ELISA) and immunogenic in mice	Expression plasmid: (pLDH-CTB-His-Term) transformation	1 mg/L culture	IMAC Purification	[107]
	*Bacillus brevis*	Affinity for GM1-ganglioside confirmed (GM1-ELISA)	Expression plasmid: (pNU212-CTB) transformation	N/A	Affinity chromatography (*D*-galactose-agarose column)	[108]
**Yeast culture**	*Pichia pastoris*	Affinity for GM1-ganglioside confirmed (GM1-ELISA) and immunogenic in mice	Expression plasmid: (pB) transformation	50 mg/L culture	IMAC Purification	[109]
**Insect cell culture**	*B. mori* (silkworm larvae)	Affinity for GM1-ganglioside confirmed (GM1-ELISA) and immunogenic in mice	Baculovirus expression system	54.4 mg/L larval hemolymph	Centrifugation	[110]
**Plants**	*Solanum tubersosum* (potato)	Affinity for GM1-ganglioside confirmed (GM1-ELISA)	Transgenic (*Agrobacterium*-mediated transformation.	0.5% of total soluble protein	Centrifugation	[112]
		Affinity for GM1-ganglioside confirmed (GM1-ELISA)	Transgenic (*Agrobacterium*-mediated transformation)	0.3% of total soluble protein	Non-purified (edible plant vaccine)	[124]
	*Daucus carota* (carrot)	Affinity for GM1-ganglioside confirmed (GM1-ELISA)	Transgenic (*Agrobacterium*-mediated transformation)	0.48% of total soluble protein	Non-purified (edible vaccine)	[113]
	*Oryza sativa* (rice seed)	Affinity for GM1-ganglioside confirmed	Transgenic (*Agrobacterium*-mediated transformation)	2.1% of total soluble protein	Non-purified (edible vaccine)	[101]
		Detected by anti-CTB antibody (Western Blot)	Transgenic (*Agrobacterium*-mediated transformation)	3.37 mg/g rice seeds	IMAC Purification	[114]
		Affinity for GM1-ganglioside confirmed	Transgenic (*Agrobacterium*-mediated transformation)	2.35 mg/g of seed	Non-purified (edible vaccine)	[115]
		Affinity for GM1-ganglioside confirmed	Transgenic (Expression plasmid biolistic-mediated transformation)	2.1% of total seed	Non-purified (edible vaccine)	[116]
	*Latuca sativa* (lettuce)	Affinity for GM1-ganglioside confirmed (GM1-ELISA)	Transgenic (*Agrobacterium*-mediated transformation)	0.24% of total soluble protein	Non-purified (edible vaccine)	[117]
	*Lycopersicon esculentum* (tomato)	Affinity for GM1-ganglioside confirmed (GM1-ELISA)	Transgenic (*Agrobacterium*-mediated transformation)	0.04% of total soluble protein	Non-purified (edible vaccine)	[118]
		Detected by anti-CTB antibody and immunogenic in mice	Transgenic (*Agrobacterium*-mediated transformation)	0.081% of total soluble protein	Non-purified (edible vaccine)	[125]
	*Nicotiana benthamiana* (a tobacco relative)	Affinity for GM1-ganglioside confirmed (GM1-ELISA) and immunogenic in mice	Transient (plant viral vectors)	1.5 mg/g leaf material or 49.9% of total soluble protein	IMAC Purification, Hydroxyapatite Chromatography (CHT column)	[100]
		Affinity for GM1-ganglioside confirmed (GM1-ELISA)	Transgenic (*Agrobacterium*-mediated transformation)	0.56% of total soluble protein	Centrifugation	[112]
		Affinity for GM1-ganglioside confirmed (GM1-ELISA)	Transgenic (*Agrobacterium*-mediated transformation)	0.095% of total soluble leaf protein	Immunoaffinity column chromatography (anti-CT IgG resin)	[119]
		Affinity for GM1-ganglioside confirmed (GM1-ELISA)	Transient (plant viral vectors)	0.14% of total soluble leaf protein	Centrifugation	[120]
	*Nicotiana tabacum* (tobacco)	Affinity for GM1-ganglioside confirmed (GM1-ELISA)	Transplastomic (Expression plasmid [pLD-LH-CTB] microprojectile bombardment)	4.1% of total soluble protein	Non-purified crude leaf extract	[121]
	*Robusta sp.* (banana callus)	Detected by anti-CT antibody (Western Blot)	Transgenic (*Agrobacterium*-mediated transformation)	125 µg/g callus tissue	Non-purified (edible vaccine)	[122]
	*Zea mays* (maize seed)	Affinity for GM1-ganglioside confirmed and immunogenic in mice	Transgenic (Plasmid microprojectile bombardment)	1.56 µg/g dry seed weight	Non-purified (edible vaccine)	[123]

* Immobilized metal ion affinity chromatography (IMAC).

A second approach is to produce rCTB in non-food or feed plants and isolate the immunogen from the tissue for vaccination. This has been undertaken in several tobacco family plants (*Nicotiana tabacum* and *N*. *benthamiana*) [100,112,119,120,121]. Daniell et al expressed rCTB in chloroplasts of transplastomic tobacco plants which enabled a high-level accumulation of glycosylation-free rCTB in leaf tissue. Alternatively, we have recently developed a transient mass production platform for a non‑glycosylated variant (Asn4→Ser) of rCTB in *N. benthamiana* using a plant virus vector system [100]. Over 1 g of the rCTB variant was produced in 1 kg of tobacco leaf (corresponding to 1000 doses of Dukoral^®^ vaccine) in 5 days post vector inoculation. The protein was efficiently purified via conventional chromatographical processes and shown to be virtually identical to original CTB in terms of physicochemical stability, GM1-ganglioside binding affinity and oral immunogenicity in mice. A major advantage to this method of production is that it is rapidly scalable based on the need for rCTB production, which could obviate the need for large vaccine stockpiling. Although the requirement of protein purification may reduce a previously conceived advantage offered by plant-based systems, it would in turn provide superior controls to the quality and dosage of vaccines and eliminate potential side effects associated with impurities.

## 5. Concluding Remarks

While first being recognized for its role in the delivery of the virulence factor of *V. cholerae*, the works highlighted in this paper show CTB’s broad utility as a cholera vaccine immunogen, vaccine adjuvant (through co-administration or conjugation), immune modulator and/or anti-inflammatory agent. This has led to the development of various rCTB expression systems in an effort to make the protein more efficient and widely available. Given that CTB appears to provide additional efficacy to killed bacteria-based cholera vaccines, development of alternative rCTB production and delivery methods may significantly contribute to cholera prevention and control. Because of the capacity to induce potent mucosal humoral immune responses, antigen-CTB fusion provides a promising strategy for vaccines against enteric pathogens and mucosally transmitted diseases. On the other hand, the immunotherapeutic potential of CTB in inflammatory diseases warrants further investigations; despite a number of studies demonstrating CTB’s anti-inflammatory effects, the underlying mechanism remains to be fully disclosed. This could be partly due to the inconsistent quality of CTB used in those studies and also attributed to different pathways altered by CTB, depending on the route/mode of administration and inflammatory conditions. Since many inflammatory diseases involve chronic and recurring inflammation, long-term immunological and toxicological impacts of repeated CTB administration need to be investigated. Nevertheless, several early-stage clinical trials have paved the way for the development of CTB-based anti-inflammatory agents. In summary, CTB has shown utility in many disease states and may ultimately be a compound with many diverse applications. The works highlighted in this paper show great promise for a single protein having multiple applications and perhaps allowing for an evolution in vaccine development.
